# In-vitro Relationship between Protein-binding and Free Drug Concentrations of a Water-soluble Selective Beta-adrenoreceptor Antagonist (Atenolol) and Its Interaction with Arsenic

**DOI:** 10.3329/jhpn.v27i1.3315

**Published:** 2009-02

**Authors:** M.A. Alam, M.A. Awal, N. Subhan, M. Mostofa

**Affiliations:** ^1^ Department of Pharmacy, Stamford University Bangladesh, 51 Siddeswari Road, Dhaka 1217, Bangladesh; ^2^ Department of Pharmacology, Bangladesh Agricultural University, Mymensingh 2202, Bangladesh; ^3^ Pharmacy Discipline, Khulna University, Khulna, Bangladesh

**Keywords:** Arsenic, Atenolol, Bovine serum albumin, Drug interactions, Plasma protein-binding

## Abstract

The degree of binding of a drug to plasma proteins has a marked effect on its distribution, elimination, and pharmacological effect since only the unbound fraction is available for distribution into extra-vascular space. The protein-binding of atenolol was measured by equilibrium dialysis in the bovine serum albumin (BSA). Free atenolol concentration was increased due to addition of arsenic which reduced the binding of the compounds to BSA. During concurrent administration, arsenic displaced atenolol from its high-affinity binding Site I, and free concentration of atenolol increased from 4.286±0.629% and 5.953±0.605% to 82.153±1.924% and 85.486±1.158% in absence and presence of Site I probe respectively. Thus, it can be suggested that arsenic displaced atenolol from its binding site resulting in an increase of the free atenolol concentration in plasma.

## INTRODUCTION

The pharmacokinetic properties of exogenous and endogenous compounds can be influenced by reversible binding to human serum albumin (HSA), which is thought to be one of the primary determinants of the pharmacokinetic properties of drugs (1-4). Therefore, when evaluating interactions among drugs, it is important to be aware of possible identities of their binding sites on the protein because any alteration in drug-binding to HSA, including binding of the antihypertensive drugs, could lead to a change in pharmacokinetic properties. Human serum albumin can be immobilized in spherical, macroporous microparticles of polyacrylamide of about 1 μm in diameter with retention of its native properties (5). Results of different experiments suggest that human serum albumin has a limited number of binding sites (4,6,7). On the basis of the probe-displacement method, there are at least three relatively-high specific drug-binding sites on the HSA molecule. These sites, commonly called warfarin, benzodiazepine, and digoxin-binding sites, are also denoted as Site I, Site II, and Site III respectively (4,8,9). It has been shown that diazepam, digitoxin, and warfarin independently bind to albumin and can conveniently be used as markers of three separate, discrete binding sites on albumin (5). Since numbers of protein-binding sites are limited, competition will exist between two drugs, and the drug with higher affinity will displace the other, causing increased free drug concentration leading to higher toxicity (10-12).

Plasma protein-binding properties are related to plasma clearance, elimination half-life, apparent volume of the distribvution, and area under the curve. The beta-blockers comprise a group of drugs that are mostly used in treating cardiovascular disorders, such as hypertension, cardiac arrhythmias, or ischemic heart diseases. As a class, the beta-blockers are quite diverse from a pharmacokinetic perspective as they display a high range of values in plasma protein-binding, percentage of drug eliminated by metabolism, or unchanged in the urine and in hepatic excretion ratio. In clinical practice, the beta-adrenergic antagonists are an extremely important class of drugs due to their high extent of use. The non-selective beta-blockers, including propranolol, oxprenolol, pindolol, nadolol, timolol, and labetalol each antagonize both ß_1_ and ß_2_-adrenergic receptors (ARs). The selective antagonists, including metoprolol, atenolol, esmelol, and acebutolol, have much greater binding-affinity for the ß_1_-AR. The selective beta-blockers are normally indicated for patients in whom ß_2_-receptor antagonism might associate with an increased risk of adverse effects. Such patients include those with asthma, or diabetes, or with peripheral vascular diseases, or Raynaud's disease (13). Of all known beta-adrenolytics, propranolol has the highest lipohilicity and can cross the blood brain barrier (BBB) whereas atenolol is a highly hydrophilic drug (partition coefficient of 0.02) (14). Hydrophilic beta-blockers, such as atenolol, are advantageous in patients who suffer from central nervous side-effects (sleep disturbances, psychosis, depression, and hallucination) during therapy with lipophilic drugs (13).

Lipophilic drugs, such as propranolol, are extensively metabolized by the liver while hydrophilic beta-blockers, including atenolol, are predominantly excreted through the kidney (Table 1) (15,16). In patients with normal renal function, the atenolol half-life was calculated to be about six hours following single 100-mg oral dose. This value increased markedly in patients with renal insufficiency, and the blood clearance of atenolol was found to have a significant correlation with the glomerular filtration rate (17).

**Table 1.** Pharmacokinetic properties of atenolol (15)

Substance	Resorption %	Bioavailability %	T_1/2_ (h)	Active metabolite	PPB %	Vd l/kg	Total clearance (mL/min)	Renal clearance (mL/min
Atenolol	50	50	6-9	--	3	0.7	100-180	100-170

min=Minute; PPB=Plasma protein-binding

Approximately 91-96% of propranolol can be bound to serum albumin or other proteins, mainly to ß_1_-acid glycoprotein and lipoproteins (18). It suggests that the lipids circulating in plasma may act as an additional depot for propanolol. Circulating free fatty acids could displace the hydrophilic drug from its protein-binding site (18,19). Contrary to propranolol, atenolol is mostly excreted through kidneys, and its elimination could not only be influenced by displacement of the drug from albumin, but also by alterations in renal blood flow in patients with hyperlipidaemia (18).

An understanding of the binding chemistry of beta-blockers, i.e. atenolol, with protein may help clinicians to interpret and predict differences among patients in pharmacologic response to these drugs. Competitive displacement is more significant when two drugs are capable of binding to the same sites on the protein. Although information resource regarding the binding of drugs to HSA is extensive, the mechanism of drug-binding to HSA is still a subject of speculation and controversy (10,11,20). Again, arsenic has a tendency to bind strongly to protein. In our rural people who are at a higher risk of arsenic ingestion in their daily water-use, the blood arsenic concentration increases (10,11). Considering this, the purpose of our work was to investigate the effect of arsenic on the concentration of the common antihypertensive drug (atenolol, a beta-blocker). Human and bovine serum albumin exhibit similar binding chemistry due to the high percentage of sequence identities between the two proteins (21). Bovine serum albumin (BSA), in lieu of HSA, was used in this study because of its low cost and easy availability.

## MATERIALS AND METHODS

### Drugs and reagents used in the experiment

Atenolol (General Pharmaceutical Ltd., Bangladesh) warfarin, diazepam, disodium hydrogen phosphate (Na_2_HPO_4_), potassium dihydrogen phosphate (KH_2_PO_4_), cellulose nitrate membrane (Medicell International Ltd., Liverpool Road, London, UK; molecular wt 1,200 Daltons), bovine serum albumin (BSA) (fatty acid-free, fraction V, molecular wt 66,500 from Sigma Chemical Ltd., USA), arsenic trioxide (As_2_O_3_), and sodium arsenate were used.

### Instruments used

The following instruments were used: p^H^ Meter (HANNA Microprocessor p^H^ Meter, Portugal), SP8-400 UV/VIS Spectrophotometer (Thermospectronic, England), Metabolic Shaking Incubator (Clifton Shaking Bath, Nical electro Ltd., England), and Micro Syringe (well. Liang. Jin. Yang.q.I., China.)

### Method used

Equilibrium dialysis was employed in the study (22,23).

### Site-specific probes method

We have used different site-specific probes to enhance our understanding of the drug-BSA interaction and thereby characterization of binding sites of the drugs used in the study on the BSA molecule (4,8,9,22,23).

Two site-specific probes were used. These are: (a) Warfarin sodium (Site I-specific probe) and (b) Diazepam (Site II-specific probe) that may be used for the identification of the binding sites of the drugs on BSA.

In the direct procedure, the ratio of BSA and probe (either warfarin or diazepam) was 1:1 (2×10^−5^ M: 2×10^−5^ M), and different concentrations of drug were added. In the reverse procedure, the ratio of BSA and drug was 1:1 (2×10^−5^ M: 2×10^−5^ M), and different concentrations of probe (Site I-specific warfarin sodium or Site II-specific diazepam) were added. After conducting equilibrium dialysis, the free concentration of probe were determined in direct procedure and reverse procedure respectively.

### Preparation of standard curve

Standard curve has been prepared using various concentrations and their corresponding concentration at pH 6.4 and 7.4. Ultraviolet (UV) spectrophotometric scanning of the drugs—atenolol, diazepam, and warfarin—showed maximum absorbance of the UV light at 275 nm, 235 nm, and 308 nm respectively. Atenolol showed linearity at a concentration range of 10-80 μM/mL with a confidence level of 0.9984 and 0.9967 at pH 6.4 and 7.4 with linear equation (Y=0.0107 X) and (Y=0.0146 X) respectively. A similar standard curve was also prepared both for diazepam and warfarin, and the concentration of those drugs were calculated using corresponding linear equations.

### Estimation of association constant of atenolol at pH 6.4 and 27 °C temperature

Estimation of the association constant of atenolol was done at pH 6.4. Ten clean and dried test tubes were taken, and 3 mL of previously-prepared 2×10^−5^ M BSA solution at pH 6.4 was taken in each of them. Aatenolol stock solution (1×10^−2^ M or 1×10^−3^ M) was added in different volumes to nine of the 10 test-tubes to have the following concentrations: 0.5×10^−5^ M, 1×10^−5^ M, 2×10^−5^ M, 3×10^−5^ M, 4×10^−5^ M, 5×10^−5^ M, 6×10^−5^ M, 7×10^−5^ M, 8×10^−5^ M, and 9×10^−5^ M. The tenth test-tube containing only BSA solution at pH 6.4 was marked as ‘control'. After mixing the solutions, these were allowed to stand for 10 minutes for maximum binding of atenolol to BSA; 2 mL from each test-tube was pipetted out and poured onto previously-prepared semi-permeable membrane-tubes and, finally, both sides of the tubes were clipped properly so that there was no leakage. The membrane-tubes containing the drug-protein mixture were immersed in ten 50-mL flasks containing 30 mL of phosphate buffer solution of pH 6.4. The mouths of the flasks were covered by foil-paper. These conical flasks were then placed in a metabolic shaker for dialysis for 10 hours at 27 °C and 20 rpm. Buffer samples were collected from each flask after complete dialysis. Free concentrations of atenolol were measured by a UV spectrophotometer at a wavelength of 275 nm.

### Estimation of association constant of atenolol at pH 7.4 and 27 °C temperature

To determine the association constant of atenolol at pH 7.4, a similar protocol as for estimation of the association constant of atenolol at pH 6.4 was followed using the buffer solution of pH 7.4. When dialysis was completed, buffer solutions were collected from each conical flask, and the free concentration of atenolol was measured by a UV spectrophotometer at a wavelength of 275 nm.

### Determination of binding site of atenolol using warfarin sodium as a Site I-specific probe

To determine the binding site of atenolol, using warfarin sodium as a Site I-specific probe, the successive steps that were followed are described below.

From the previously-prepared 2×10^−5^ M BSA solution, 3 mL was taken in each of the eight cleaned and dried test-tubes; 1×10^−3^ M warfarin solution was added to seven of the eight test-tubes, and the final ratio of protein and warfarin was 1:1 (2×10^−5^ M: 2×10^−5^ M) in each of these seven test-tubes. The eighth test-tube containing only BSA solution was marked as ‘blank' or ‘control'. These mixtures were allowed to stand for 10 minutes for allowing binding of the warfarin to its particular binding site. Atenolol solutions (either 2×10^−2^ M or 2×10^−3^ M) were added with increasing concentrations into six of the seven test-tubes containing 1:1 mixture of protein-warfarin. The final ratios of protein: warfarin: atenolol were 1:1:0, 1:1:1, 1:1:2, 1:1:3, 1:1:4, 1:1:5, and 1:1:6. The remaining test-tube contained only protein-warfarin mixture (1:1). After pipetting, the solution was properly mixed and allowed to stand for 10 minutes to ensure maximum binding of atenolol to Site I and thereby displacing the probe from Site I on BSA. From each test-tube, 2-mL solution was taken into eight different semi-permeable membrane-tubes. The two ends of the membrane-tubes were clipped to ensure that there was no leakage, and the rest of the experiment was done as described above using phosphate buffer solution of pH 7.4.

### Determination of binding site of atenolol using diazepam as a Site II-specific probe

To perform the experiment, the previously-described procedure has been followed successively using diazepam solution. The final ratios of protein: diazepam: atenolol were 1:1:0, 1:1:1, 1:1:2, 1:1:3, 1:1:4, 1:1:5, 1:1:6, and 1:1:9. Atenolol was not present in the first test-tube which contained only protein-diazepam mixture (1:1). At the end of dialysis, samples were collected from each flask. The free concentrations of diazepam were measured using a UV spectrophotometer at a wavelength of 235 nm (BP). Reverse experiment was also being conducted followed by a similar protocol by adding diazepam in an increasing concentration and measuring the free atenolol concentration by spectrophotometer.

### Effect of arsenic on atenolol-binding to BSA in presence of Site I-specific probe warfarin-sodium

From the previously-prepared 2×10^−5^ M BSA solution and 1×10^−2^ M warfarin solution, 2 mL and 12 mL, respectively, were added to each of the seven cleaned and dried test-tubes. The final ratio between protein and warfarin was 1:1 (2×10^−5^ M: 2×10^−5^ M) in each of seven test-tubes so that Site I is sufficiently blocked by warfarin-sodium. The seventh test-tube containing only BSA solution was marked as blank. After that, atenolol was added in six of the seven test-tubes, and protein-warfarin-atenolol ratio was 1:1:1 (2×10^−5^ M:2×10^−5^ M:2×10^−5^ M). Arsenic was added with an increasing concentration into five of the six test-tubes containing 1:2:1 mixture of protein-warfarin-atenolol to make the final ratio of protein-warfarin-atenolol arsenic 1:2:1:0, 1:2:1:1, 1:2:1:2, 1:2:1:4, 1:2:1:6, 1:2:1:8, 1:2:1:10. Arsenic was not added to one test-tube. The solutions were then properly mixed and allowed to stand for 15 minutes for the confirmation of maximum binding to BSA. After that, the solution was pipetted out and poured into seven semi-permeable membrane-tubes. The two ends of the membrane-tubes were clipped to ensure that there was no leakage, and the rest of the experiment was done as described above using phosphate buffer solution of pH 7.4.

### Effect of arsenic on atenolol-binding to BSA in absence of Site I-specific probe warfarin-sodium

To perform the experiment, the previously-des-cribed procedure was followed successively in absence and presence of warfarin sodium. Arsenic was added with an increasing concentration to five of the six test-tubes containing 1:1 mixture of protein-atenolol to make the final ratio of protein:atenolol: arsenic 1:1:0, 1:1:1, 1:1:2, 1:1:4, 1:1:6, 1:1:8, 1:1:10. Arsenic was not added to the first test-tube which contained only protein-atenolol mixture (1:1). At the end of dialysis, samples were collected from each flask. The free concentrations of atenolol were measured by a UV spectrophotometer at a wavelength of 275 nm (BP).

## RESULTS

Both association constant (k_a_) and number of binding sites (n) of atenolol were determined using the Scatchard plot. To estimate the binding parameters of atenolol, equilibrium dialysis (ED) was used and the subsequent non-linear shape of the Scatchard plot describes both high- and low-affinity binding sites of drugs on protein molecule.

### Determination of association constant and number of binding site

Atenolol was characterized by a high-affinity association constant (k1) to BSA and the value at pH 7.4 was (4.6±0.151)×10-5 M (Table 2) while the low-affinity association constant (k2) for atenolol was found to be (3.03±0.086)×10-5 M. For this drug, the number of high-affinity and low-affinity binding sites was (0.61±0.199) ×10-5 M and (1.33±0.114) ×10-5 M respectively at pH 7.4. In the case of atenolol bound to BSA, the high-affinity association constant (k1) was found to increase when pH was changed from 7.4 to 6.4, and the value for k1 and k2 was (5.06±0.202) ×10-5 M and (4.23±0.159) ×10-5 M respectively (Table 2). As a consequence for this drug, the number of high-affinity and low-affinity binding sites was (0.81±0.04) ×10-5 M and (2.03±0.201) ×10-5 M respectively at pH 6.4. When the changes of physiological pH occur, BSA undergoes conformational alteration, which is generally termed N-B transition. BSA remains almost entirely in neutral form at pH 6 and in basic form at pH 9. When the protein is in the B-conformation, fewer protons are bound to BSA than that in the N-conformation. Thus, the high-affinity and low-affinity binding of atenolol is affected by change in pH. These differences in effect of pH may be due to the structural modification of protein molecule and, for this reason, at a given pH value, the binding site for atenolol is more suitable or properly accommodated, while at other pH values, the binding sites become less convenient and less accommodating to the drugs in concern.

**Table 2.** Parameters of atenolol bound to BSA at different pH values

pH	Association constant		No. of binding sites	
	K_1_ (high affinity) × 10^−5^ M	K_2_ (low affinity) × 10^−5^ M	n_1_ (high affinity) × 10^−5^ M	n_2_ (low affinity) × 10^−5^ M
6.4	5.06±0.202	4.23±0.159	0.81±0.04	2.03±0.201
7.4	4.6±0.151	3.03±0.086	0.61±0.199	1.33±0.114

Values represent three consecutive experiments and expressed as mean±standard error of mean

### Determination of binding site

**Fig. 1.**
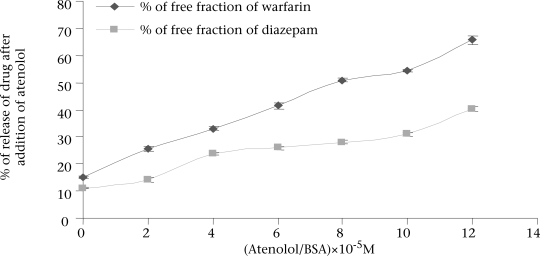
Free concentration of warfarin and diazepam bound to BSA upon the addition to atenolol at pH 7.4 and 27 °C

**Fig. 2.**
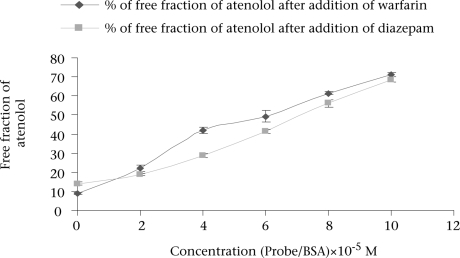
Free concentration of atenolol when used with warfarin and diazepam bound to BSA at pH 7.4 and at temperature of 27 °C

Well-established probes, which are specific for particular sites on the albumin molecule, are used for the identification of the binding site of the drugs on the protein molecule. If a drug is able to displace a probe from its binding site, it is assumed that the drug also binds to that particular site. Thus, the binding site and specificity and relative strength of binding to albumin of atenolol have been determined by this principle. Here, as Site I-specific probe, warfarin sodium and as Site II-specific probe, diazepam, were used. To characterize the binding site of atenolol, the free concentration of warfarin-sodium (Site I-specific probe) bound to BSA was measured upon the addition of atenolol. It was found that the free concentration of warfarin-sodium increased from 14.966±0.351% (as % of initial) to 65.66±1.457% when the ratio of atenolol to BSA was increased to 6 (Fig. 1). In contrast, under the same experiment conditions when, in lieu of warfarin-sodium, diazepam was used as Site II-specific probe, the increment of the free concentration of diazepam by atenolol was from 11.166±0.152% (as % of initial) to 40.1±1.014% (Fig. 1). From these data, this is evident that, upon addition of atenolol, the increment of free concentration of warfarin-sodium is obviously greater than that of diazepam. Thus, it can be concluded that atenolol preferentially binds to Site I. Again, as the displacement of diazepam is quite pronounced, it can be also suggested that atenolol, in addition to Site I, also binds to Site II on the BSA molecule but to a lower extent. In the reverse experiment, the free concentration of atenolol was increased from 8.7±0.529% (as % of initial) to 71.133±1.059% when warfarin to BSA ratio was 6 (Fig. 2). On the other hand, the free concentration of atenolol was increased from 13.933±0.702% (as % of initial) to 68.65±1.261% at the ratio of diazepam to BSA were also 6 (Fig. 2). From these data, it is clear that the increment of atenolol due to displacement by warfarin (Site I probe) is higher than that of atenolol when displaced by diazepam. Thus, the findings of the reverse experiment also were in agreement with the findings of the previous experiment.

Values represent three consecutive experiments and expressed as mean±standard deviation; warfarin 2×10^−5^ M, diazepam 2×10^−5^ M, BSA 2×10^−5^ M, and atenolol 0–12×10^−5^ M were used

Values represent three consecutive experiments and expressed as mean±standard deviation; warfarin 0–12×10^−5^ M, diazepam 0–12×10^−5^ M, BSA 2×10^−5^ M, and atenolol 2×10^−5^ M were used

### Displacement of atenolol due to the effect of arsenic

**Fig. 3.**
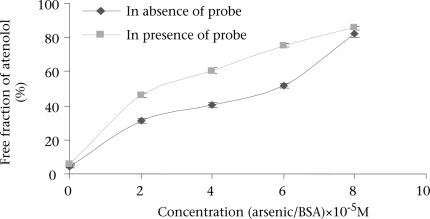
Free concentration of atenolol bound to BSA upon the addition of arsenic trioxide in absence and presence of Site I-specific probe

**Fig. 4.**
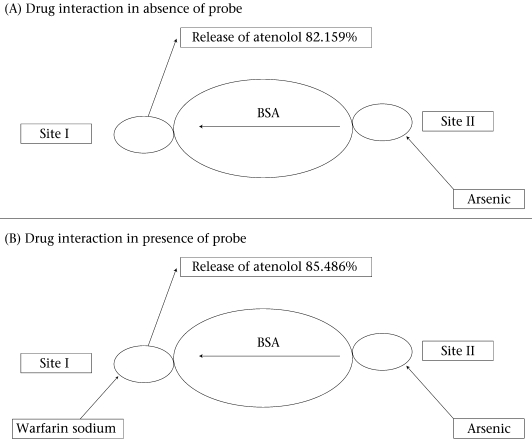
Proposed models of the atenolol-BSA-arsenic interaction in absence and presence of warfarin (Site I-specific probe)

During concurrent administration of atenolol and arsenic, site-to-site displacement takes place, and arsenic displaced atenolol from its binding sites (Fig. 3). In presence of probe, free concentration of atenolol was more prominent. This displacement may be due to reduction of the binding site on bovine serum albumin. As observed from the model (Fig. 4), during concurrent administration, arsenic displaced atenolol from its high-affinity binding Site I. Thus, free concentration of atenolol increased from 4.286±0.629% and 5.953±0.605% to 82.153±1.924% and 85.486±1.158% in absence and in presence of Site I probe respectively.

Values represent three consecutive experiments and expressed as mean± standard deviation; warfarin 2×10^−5^ M, arsenic 0–12×10^−5^ M, BSA 2×10^−5^ M, and atenolol 2×10^−5^ M were used

Concentrations used: [BSA]=[Atenolol]=2×10^−5^ M [Arsenic]=0–12×10^−5^ M, [Warfarin]=2×10^−5^ M

## DISCUSSION

In-vitro binding of water-soluble selective beta-adrenergic-blocker drug with bovine serum albumin was studied, and a lower association constant at pH 6.4 compared to pH 7.4 was found with a limited number of corresponding binding sites. HSA is an abundant transport-protein found in plasma which binds a wide variety of drugs in two primary binding sites (I and II) that can have a significant impact on their pharmacokinetics (24). Site-specific displacement interaction also provides information about the binding site. Atenolol substantially binds with Site I of BSA but also binds with Site II to a lesser extent. In a previous experiment, it was observed that the binding of arsenic to BSA occurred at Site II (9). The displacement interaction will be prominent for the binding, occurring at the same site. It should also be accountable for the binding to opposite site as these two binding sites exist in a transitional state.

Protein-binding of some beta-blockers was determined in-vitro using equilibrium dialysis of labelled drug. Oxprenolol and propranolol were highly bound to serum, alprenolol, and pindolol, but timolol to a lesser degree, and atenolol, metoprolol, and sotalol were negligibly bound (25). Binding studies with beta-adrenoceptor-blocking drugs in the presence of human plasma in combination with investigations of the sympatholytic activity in volunteers have shown that there is a correlation between plasma levels and drug action (26). This is of major importance for the duration of action which depends on dose and plasma half-life. These observations may be of clinical significance as patients with coronary heart disease, who take their drug once a day in the morning, need protection for the rest of the day. It is known that the incidence of silent ischaemic episodes, myocardial infarctions, and strokes increase in the early morning hours. Therefore, a preferential drug would guarantee a full therapeutic effect for 24 hours. ß-adrenoreceptor antagonists improve acute outcomes and long-term prognosis in ischaemic heart disease (27) and reduce peri-operative events among high-risk patients undergoing major non-cardiac and vascular surgery (28,29). The beta-adrenergic receptor is a glycoprotein (30). ß_2_-AR contributes to cardiovascular regulation by influencing several functions, and results of previous studies suggest that a decreased function of the ß_2_-AR may be involved in essential hypertension. ß_2_-AR is polymorphic, and certain polymorphisms of these receptors are of functional importance (31). Stimulation of the ß_1_-AR in ß_2_-AR-KO myocytes produces the greatest increase in contraction rate through a signalling pathway that requires protein kinase A (P_K_A) activation. In contrast, stimulation of the ß_2_-AR in ß_1_-AR-KO myocytes results in a biphasic effect on contraction rate with an initial increase in rate that does not require P_K_A, followed by a decrease in rate that involves coupling to a pertussis toxin-sensitive G protein (32). The beta-adrenergic receptor kinase (ß-ARK) specifically phosphorylates the agonist-occupied form of the beta-adrenergic and related G protein-coupled receptors (33). Arsenic-induced decreased expression of ß_2_-AR in cultured keratinocytes has also been reported (34). Recent studies of the effects of arsenic on cardiac electrophysiology have focused on arsenic trioxide (As_2_O_3_). The interest in studying arsenic trioxide is related to its use in the treatment of cancer, and some indications of heart problems associated with the treatment (35) reported that arsenic trioxide prolongs the duration of AP.

Ingested inorganic arsenic has been known to be related to the development of peripheral vascular disease and ischaemic heart disease among residents consuming water with high arsenic concentrations in Taiwan (36-39), Chile (40-42), and Mexico (43). A comparison of the prevalence of hypertension among subjects with and without arsenic exposure through drinking-water was done in Bangladesh. This study showed a dose-response relationship between inorganic arsenic exposure from drinking-water and risk of hypertension (44). The effect of long-term exposure to inorganic arsenic on the development of hypertension has rarely been studied, and the mechanism for inorganic arsenic to induce hypertension remains unclear (45). Long-term arsenic exposure has been documented to be associated with the development of peripheral neuropathy. Characteristic electromyographic changes include decreased nerve-conduction amplitude, with little changes in nerve-conduction velocity (46). Arsenic neuropathy has been classified as a distal axonopathy with axonal degeneration, especially of large myelinated fibres of both sensory and motor neurons (47).

A target of arsenite in liver-cells may be the P_450_s, which are gene families of haemoproteins that catalyze the oxidation of many endogenous and exogenous lipophilic chemicals (48). Earlier investigations revealed that severe exposure to arsenite reduces both basal and induced levels of some hepatic P_450_s as measured spectrally or enzymatically (49,50) in rodents, and in primary cultures of rat hepatocytes, it has also been found to decrease induction of CYP_1_A_1/2_, 2B1, and 3A23 (51). The effect of arsenite to decrease P_450_s may cause major implications in human health by altering the metabolism and elimination of toxic chemicals and drugs that are substrates for such P_450_s. CYP3As are the most abundant P_450_ proteins found in the human liver, accounting for between 30% and 60% of the total cytochrome P_450_ content, with CYP3A4 being the major hepatic CYP3A present (52). In humans, CYP3A proteins are involved in the metabolism of 45% to 60% of all currently-used drugs (53,54). Therefore, variability in CYP3A4 enzyme system expression would be expected to have a profound effect on the efficacy and safety of drugs that have a narrow therapeutic index and are metabolized by CYP3As.

It was previously reported that 5 μM of arsenite not only decreases phenobarbital (PB)-induced CYP3A23 protein, with a little to no decrease in CYP3A23 mRNA, in primary cultures of rat hepatocytes (51) but also decreases CYP1A1 and CYP1A2 expression in primary human hepatocytes (55), benzo[*a*]pyrene-induced CYP1A1 and CYP1B1 expression in T-47D human breast-cancer cells (56), and benzo[*k*]-fluoranthene-mediated induction of CYP1A1 mRNA in HepG2 cells (57). Although the mechanisms underlying arsenite-mediated decrease in P_450_s have yet to be identified, several hypotheses have been generated, including transcriptional and post-translational events (56,58).

Arsenic has been reported to induce chronic renal insufficiency from cortical necrosis (59), and signs of arsenic-induced renal injury include haematuria, leukocyturia, and glycosuria (60). The arsenic-related hypertension observed in that study is the result of possible neurological, and renal defects induced by arsenic need further exploration. Treatment for hypertension with beta-blockers in the arsenic-affected area where people are bound to drink arsenic-containing well-water daily should require additional precautions. Atenolol does not undergo hepatic metabolism and has rarely been associated with liver injury. Arsenic may cause renal insufficiency which could lead to accumulation of more atenolol in plasma because excretion of atenolol is totally dependent on renal function and increases its plasma half-life. Moreover, as shown in the present study, arsenic displaces the in-vitro atenolol binding to BSA and increases the free concentration of drug which may be available for further action upon the beta-receptor. Further research is needed to elicit the exact mechanism of drug interaction with arsenic and its binding with bovine serum albumin.
